# Protective Effects of Platycodin D on Lipopolysaccharide-Induced Acute Lung Injury by Activating LXRα–ABCA1 Signaling Pathway

**DOI:** 10.3389/fimmu.2016.00644

**Published:** 2017-01-03

**Authors:** Xiaoyu Hu, Yunhe Fu, Xiaojie Lu, Zecai Zhang, Wenlong Zhang, Yongguo Cao, Naisheng Zhang

**Affiliations:** ^1^Department of Clinical Veterinary Medicine, College of Veterinary Medicine, Jilin University, Changchun, China

**Keywords:** LXRα, ABCA1, TLR4, lipid raft, A549 lung epithelial cells

## Abstract

The purpose of this study was to investigate the protective effects of platycodin D (PLD) on lipopolysaccharide (LPS)-induced acute lung injury (ALI) and clarify the possible mechanism. An LPS-induced ALI model was used to confirm the anti-inflammatory activity of PLD *in vivo*. The A549 lung epithelial cells were used to investigate the molecular mechanism and targets of PLD *in vitro*. *In vivo*, the results showed that PLD significantly attenuated lung histopathologic changes, myeloperoxidase activity, and pro-inflammatory cytokines levels, including TNF-α, IL-1β, and IL-6. *In vitro*, PLD inhibited LPS-induced IL-6 and IL-8 production in LPS-stimulated A549 lung epithelial cells. Western blot analysis showed that PLD suppressed LPS-induced NF-κB and IRF3 activation. Moreover, PLD did not act though affecting the expression of TLR4. We also showed that PLD disrupted the formation of lipid rafts by depleting cholesterol and prevented LPS-induced TLR4 trafficking to lipid rafts, thereby blocking LPS-induced inflammatory response. Finally, PLD activated LXRα–ABCA1-dependent cholesterol efflux. Knockdown of LXRα abrogated the anti-inflammatory effects of PLD. The anti-inflammatory effects of PLD was associated with upregulation of the LXRα–ABCA1 pathway, which resulted in disrupting lipid rafts by depleting cholesterol and reducing translocation of TLR4 to lipid rafts.

## Introduction

Acute lung injury (ALI) takes responsibility for significant morbidity and is associated with an up to 30–50% mortality rate (1). The epithelial cells played an important role in innate immune responses. It not only provides a physico-chemical barrier but also responds to inhaled microorganisms by releasing inflammatory mediators (2). Lipopolysaccharide (LPS) activates TLR4 signaling pathway and triggers an inflammatory response in ALI (3). ABCA1 is a lipid pump that effluxes cholesterol and phospholipid out of cells. Activation of ABCA1 and ABCG1 could induce cholesterol efflux from plasma membrane microdomains known as lipid rafts (4). Lipid rafts, enriched in cholesterol and sphingolipids, are microdomains of the plasma membrane. They serve as a platform for signal transduction and play an important role in TLR4 signal pathway (5). Treatment with raft-disrupting drugs could suppress LPS-induced NF-κB activation and TNF-α production (6, 7).

Platycodin D (PLD), the major triterpene saponin in the root of *Platycodon grandiflorum*, has been reported to have various pharmacological activities. PLD exhibited a broad spectrum anti-inflammatory effect. PLD could inhibit LPS-induced TNF-α and IL-1β production in RAW264.7 cells (8). PLD could inhibit NF-κB activation (9). Furthermore, PLD was found to attenuate airway inflammation by inhibiting NF-κB activation (10). However, the effects of PLD on LPS-induced ALI and the molecular targets remain unclear. The purpose of this work was to examine the anti-inflammatory effects of PLD in LPS-induced ALI and to identify the molecular targets of PLD in A549 lung epithelial cells.

## Materials and Methods

### Materials

Platycodin D was purchased from the National Institute for the Control of Pharmaceutical and Biological Products (Beijing, China). Dimethyl sulfoxide (DMSO), LPS (*Escherichia coli* 055:B5), and 3-(4,5-dimethylthiazol-2-y1)-2,5-diphenyltetrazolium bromide (MTT) were purchased from Sigma Chemical Co. (St. Louis, MO, USA). Human TNF–α, IL-6, IL-1β, and IL-8 ELISA kits were purchased from Biolegend (CA, USA). Human mAb Phospho-NF-κB and human mAb NF-κB, human mAb Phospho-IRF3, and mAb IRF3 were purchased from Cell Signaling Technology Inc. (Beverly, MA, USA). β-actin was purchased from Tianjin Sungene Biotech Co. Ltd. (Tianjin, China). All other chemicals were of reagent grade.

### Animals and Treatment

All animal experiments were performed in accordance with the guide for the Care and Use of Laboratory Animals published by the US National Institute of Health. Male BALB/c mice (8–12 weeks), which weighed 18–20 g, were purchased from the Center of Experimental Animals of Baiqiuen Medical College of Jilin University (Jilin, China). All animals were housed in microisolator cages and received food and water *ad libitum*. Laboratory temperature was 24 ± 1°C, and relative humidity was 40–80%. Before experimentation, mice were housed for a minimum of 4–6 days to adapt them to the environment.

All mice were randomly divided into the following groups: control group, LPS group, PLD (20, 40, and 80 mg/kg) + LPS groups, and dexamethasone (DEX) + LPS group. PLD and DEX were intraperitoneally injected before stimulation with LPS. Meanwhile, control and LPS groups were administrated with an equal volume of distilled water. One hour later, 10 µg of LPS in 50 µl of PBS was introduced intranasally (i.n.) to produce ALI. Control mice received 50 µl PBS. Mice were humanely sacrificed 7 h after LPS stimulation. Mice all underwent combined with tracheal intubation and bronchoalveolar lavage fluid (BALF) were repeatedly collected for analysis.

### Histopathologic Evaluation of the Lung Tissue

Histopathologic examination was performed on mice that were not subjected to BALF collection. Seven hours after LPS treatment, lungs tissues were collected and fully fixed with 10% buffered formalin for approximately 1 week. The lung tissues were embedded in paraffin and cut into 5 µm sections, followed by hematoxylin and eosin (H&E) staining. Pathological changes in the lungs were examined with light microscope (Olympus, Japan).

### Inflammatory Cell Counts of BALF

The BALF samples were collected and centrifuged at 3,000 rpm for 10 min at 4°C. The cells were resuspended in PBS for the total cell counts using a hemacytometer, and cytospins were prepared for differential cell counts by staining with the Wright–Giemsa staining method (11).

### Pulmonary Myeloperoxidase (MPO) Activity in ALI Mice

The MPO activity in homogenates of lung tissues was evaluated using MPO test kits (Nanjing Jiancheng Bioengineering Institute). Briefly, the mice were killed 7 h after treatment with LPS. The lung tissues were collected to make 5% homogenate with extraction buffer, followed by constitute with 0.9 ml homogenate and 0.1 ml reaction buffer was hated to 37°C in water for 15 min. The enzymatic activity was tested by automatic microplate reader (Tecan Sunrise) in absorbance at 460 nm.

### Cell Culture and Treatment

The human lung epithelial cell line A549 was obtained from American Type Culture Collection (ATCC, Manassas, VA, USA) and maintained in DMEM supplemented with 10% fetal bovine serum (FBS) at 37°C with 5% CO_2_. HEK293T cells, purchased from ATCC (Manassas, VA, USA), were cultured in DMEM (Invitrogen) containing 10% FBS at 37°C with 5% CO_2_.

### Cell Viability Assay

The cell viability was measured by MTT assay. A549 cells were plated at a density of 4 × 10^5^ cells/ml in 96-well plates for 1 h, then the cells were treated with 50 µl of PLD at different concentrations (0–20 µM) for 1 h, followed by stimulation with 50 µl LPS. After 18 h of LPS stimulation, 20 µl MTT (5 mg/ml) was added to each well, and the cells were further incubated for an additional 4 h. The supernatant was removed and the formation of formazan was resolved with 150 μl/well of DMSO. The optical density was measured at 570 nm on a microplate reader (TECAN, Austria).

### ELISA Assay

The A549 lung epithelial cells were treated with or without PLD (5, 10, and 20 µM). One hour later, the cells were treated with LPS for another 24 h. The sample was centrifuged (2,000 rpm for 40 min at 4°C), and supernatant was collected to measure the levels of cytokines IL-6 and IL-8 by ELISA according to the manufacturer’s protocols (BioLegend). Read plate at 570 nm by automatic microplate reader (Tecan Sunrise).

### Western Blot Analysis

Total proteins within cells were extracted using the M-PER Mammalian Protein Extracted Reagent (Thermo). The concentration of protein was measured through a BCA protein assay kit (Pierce, Rockford, IL, USA). Lysate samples which contain same amounts of protein (40 µg) were fractionated by SDS-PAGE and then transferred onto a PVDF membrane. We blocked the membrane with 5% skimmed milk at room temperature continuing for 2 h, which was as well probed with primary antibodies (1:1,000 dilutions in TBS-T) at 4°C overnight. The membrane was washed with PBS-T for three times. After that, another 1 h was needed to incubate the membrane with specific secondary antibody at room temperature. The targeted proteins could be seen with Supersignal West Pico Chemiluminescent Substrate (Thermo Scientific, USA). The β-actin protein played a role as an internal control. The density of each band was quantified using image analysis software (Image J). The protein levels were normalized to β-actin.

### Immunocytochemistry and Lipid Rafts Staining

Cell membrane lipid rafts were labeled with choleratoxin subunit B (CTxB). Cells were fixed in 4% formaldehyde for 40 min at room temperature. Then, it was incubated with Alexa Fluor 488-conjugated CTxB (5 µg/ml) for 20 min and washed with PBST three times. Cells were stained with Hochest for 5 min. The fluorescent images were obtained using scanning confocal microscope (Olympus FluoView FV1000).

### Isolation of Lipid Rafts

Lipid rafts were isolated, as described previously (12). Briefly, A549 cells were lysed in ice-cold MBS buffer (25 mM MES, pH 6.5, 150 mM NaCl, 1 mM Na_3_VO_4_, 1% Triton X-100, and protease inhibitors). Lysates were mixed with 4 ml of 40% sucrose by mixing with 2 ml of 80% sucrose and overlaid with 4 ml of 35% sucrose and 4 ml of 5% sucrose in MBS buffer. Samples were ultracentrifuged at 39,000 rpm for 18 h and fractionated into 12 subfractions.

### Quantification of Cholesterol Levels in Lipid Rafts of A549 Cells

Lipid rafts were isolated as described above. Cholesterol level of lipid raft was assayed by gas–liquid chromatography, as previously described (13).

### Cholesterol Replenishment Experiment

A549 cells were treated with culture medium alone or medium containing PLD (3, 6, and 12 µM), or MβCD (10 mM) at 37°C for 60 min. Subsequently, the cells were washed with PBS and incubated 30 min with medium or medium containing water-soluble cholesterol (84 µg/ml). Then, the cells were treated with LPS. The effect of PLD on LPS-induced cytokines was detected, as mentioned above.

### LXR Receptor Gene Assay

For LXR activation assay, A549 cells were transfected with 0.75 µg of LXRE-driven luciferase reporter vector (LXRE-tk-Luc) and 0.75 µg of β-galactosidase control vector (Promega) using FuGENE HP transfection reagent. Six hours later, A549 cells were incubated with PLD for 12 h. The β-galactosidase enzyme activity was measured using the β-galactosidase Enzyme System (GloMax-96 Microplate Luminometer, Promega).

### Transient Transfection of siRNA against LXRα

siRNA against LXRα (si-LXRα; ON-TARGET plus SMART pool), non-targeting siRNA (si-control), and the DharmaFECT transfection reagent was purchased from Thermo Scientific Dharmacon (USA). Si-LXRα and si-control stock solutions (20 µM) were solved with DEPC water to form 5 µM solutions. When the cells reached to 60% confluency, the transfection reagent was mixed with 5 µM si-LXRα or si-control for 20 min. Then, it was added to the medium to reach the concentration of 25 µM. The A549 cells were treated with siRNA for 48 h. The effects of siRNA on LXRα were detected by western blot analysis.

### Statistical Analysis

All values are expressed as the means ± SEM. Differences between groups were analyzed using a one-way ANOVA (Dunnett’s *t*-test) and a two-tailed Student’s *t*-test. The results were considered statistically significant at *p* < 0.05 or *p* < 0.01.

## Results

### Effects of PLD on LPS-Mediated Lung Histopathologic Changes

**Figure 1:**
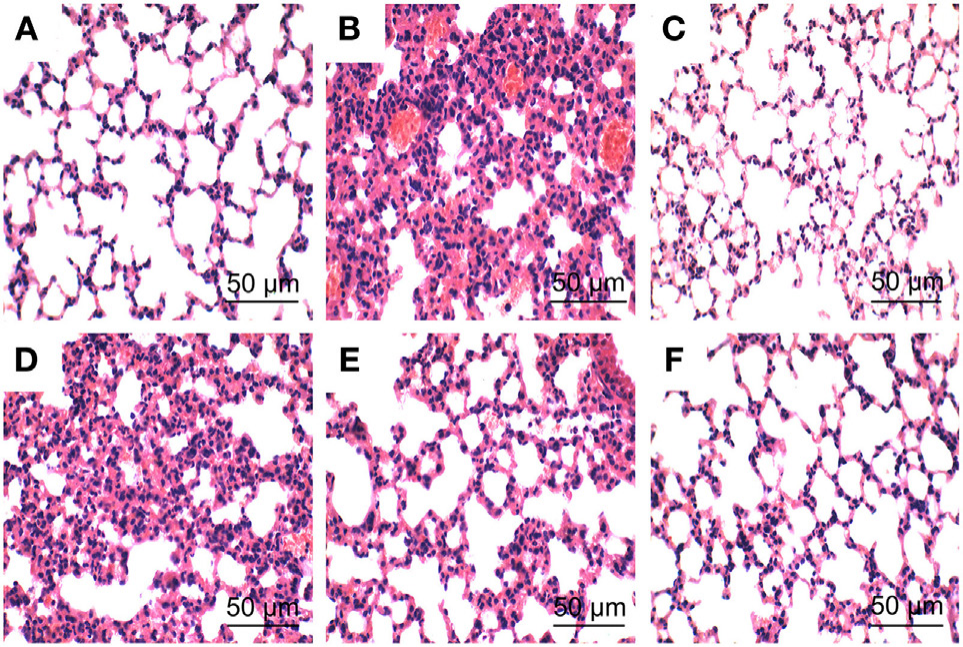
**Histopathologic sections of lung tissues (HE, ×100), lung tissue of control group (A), lipopolysaccharide (LPS) group (B), LPS + DEX group (C), LPS + platycodin D (PLD) 20 mg/kg (D), LPS + PLD 40 mg/kg (E), and LPS + PLD 80 mg/kg (F)**.

To investigate the protective effects of PLD on LPS-induced ALI, lung pathologic changes were determined by H&E staining. As shown in Figure 1, lung tissues from the control group showed a normal structure and no histopathologic changes under a light microscope (Figure 1A). While, lung of mice administered by LPS showed several obvious inflammatory histological changes, such as pulmonary congestion, focal area of fibrosis with collapse of air alveoli and emphysematous, thickening of the alveolar wall, and areas of inflammatory infiltration (Figure 1B). However, LPS-induced pathological changes were significantly attenuated by PLD (20, 40, and 80 mg/kg) (Figures 1D–F) and DEX (5 mg/kg) treatment (Figure 1C).

### Effects of PLD on Inflammatory Cell Count in the BALF of LPS-Induced ALI Mice

**Figure 2:**
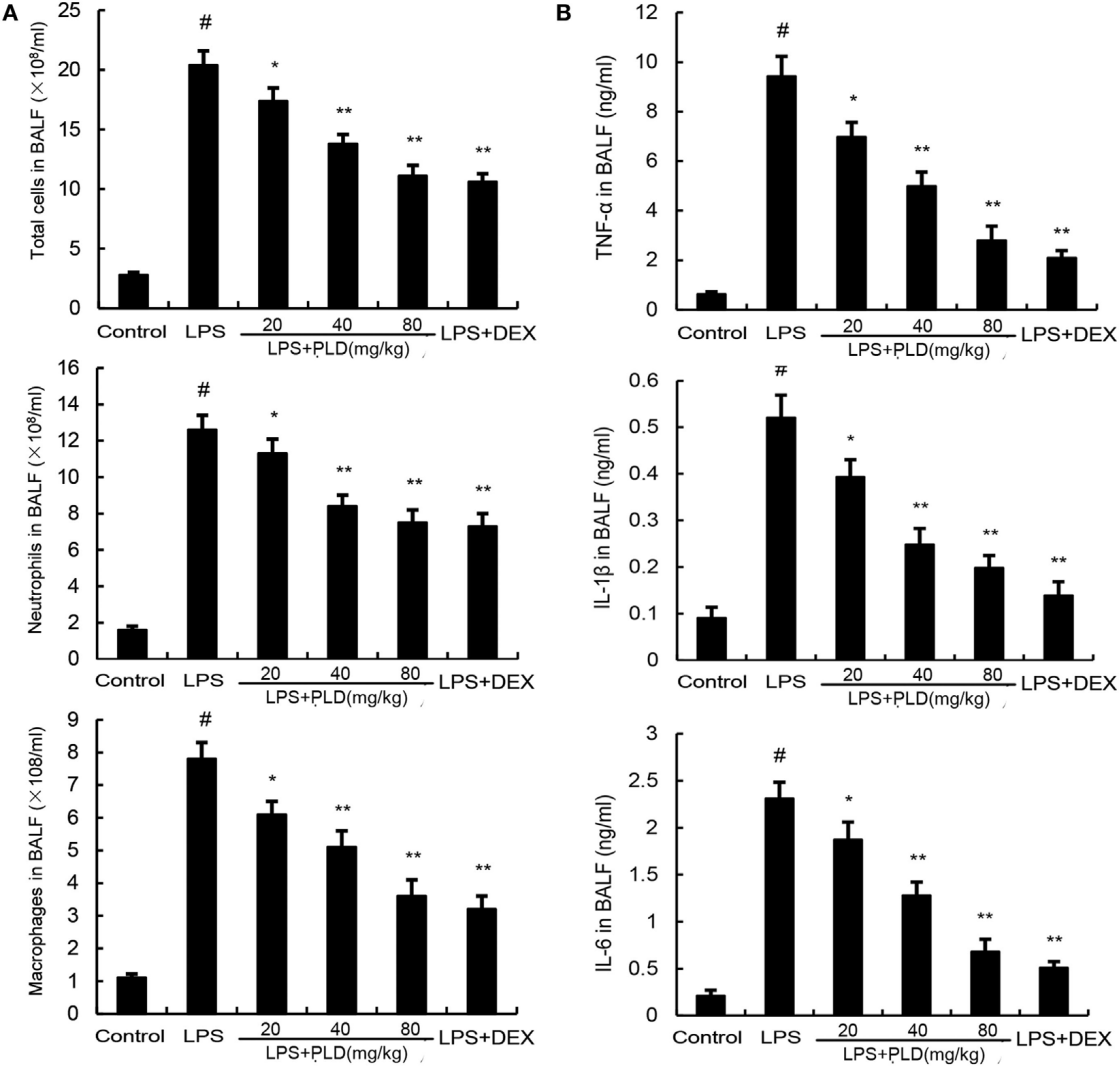
**(A)** Effects of platycodin D on the number of total cells, neutrophils, and macrophages in the BALF of lipopolysaccharide (LPS)-induced ALI mice. BALF was collected at 7 h after LPS administration to measure the number of total cells, neutrophils, and macrophage. **(B)** The levels of TNF-α, IL-1β, and IL-6 in the BALF. Data are presented as mean ± SEM (*n* = 12 in each group) and differences between mean values were assessed by one-way ANOVA (Dunnett’s *t*-test) and the two-tailed Student’s *t*-test. ^#^*p* < 0.01 significantly different from control group; *p < 0.05 and **p < 0.01 significantly different from LPS group.

To investigate the effects of PLD on inflammatory cell infiltration, the numbers of inflammatory cells, such as neutrophils and macrophages, in BALF were analyzed by Wright–Giemsa staining method. As shown in Figure 2A, LPS challenge significantly increased the number of total cells, neutrophils and macrophages compared with the control group (*p* < 0.01). Meanwhile, pretreatment with PLD (20, 40, and 80 mg/kg) and DEX (5 mg/kg) was found to significantly decrease the number of total cells (*p* < 0.01), neutrophils (*p* < 0.01), and macrophages (*p* < 0.01).

### Effects of PLD on Cytokine Production in the BALF of LPS-Treated ALI Mice

To investigate the anti-inflammatory effects of PLD, we measured changes in inflammatory cytokines TNF-α, IL-1β and IL-6 in BALF. As shown in Figure 2B, stimulation with LPS caused a noticeable lift of TNF-α (*p < 0.05), IL-1β (*p < 0.05 or **p < 0.01), and IL-6 (*p < 0.05 or **p < 0.01) compared with control group, while the one with pretreatment of PLD (20, 40, and 80 mg/kg) and DEX, it is the number of TNF-α, IL-1β, and IL-6 induced by LPS is dependently suppressed.

### Effects of PLD on the MPO Activity in ALI Mice Induced by LPS

**Figure 3:**
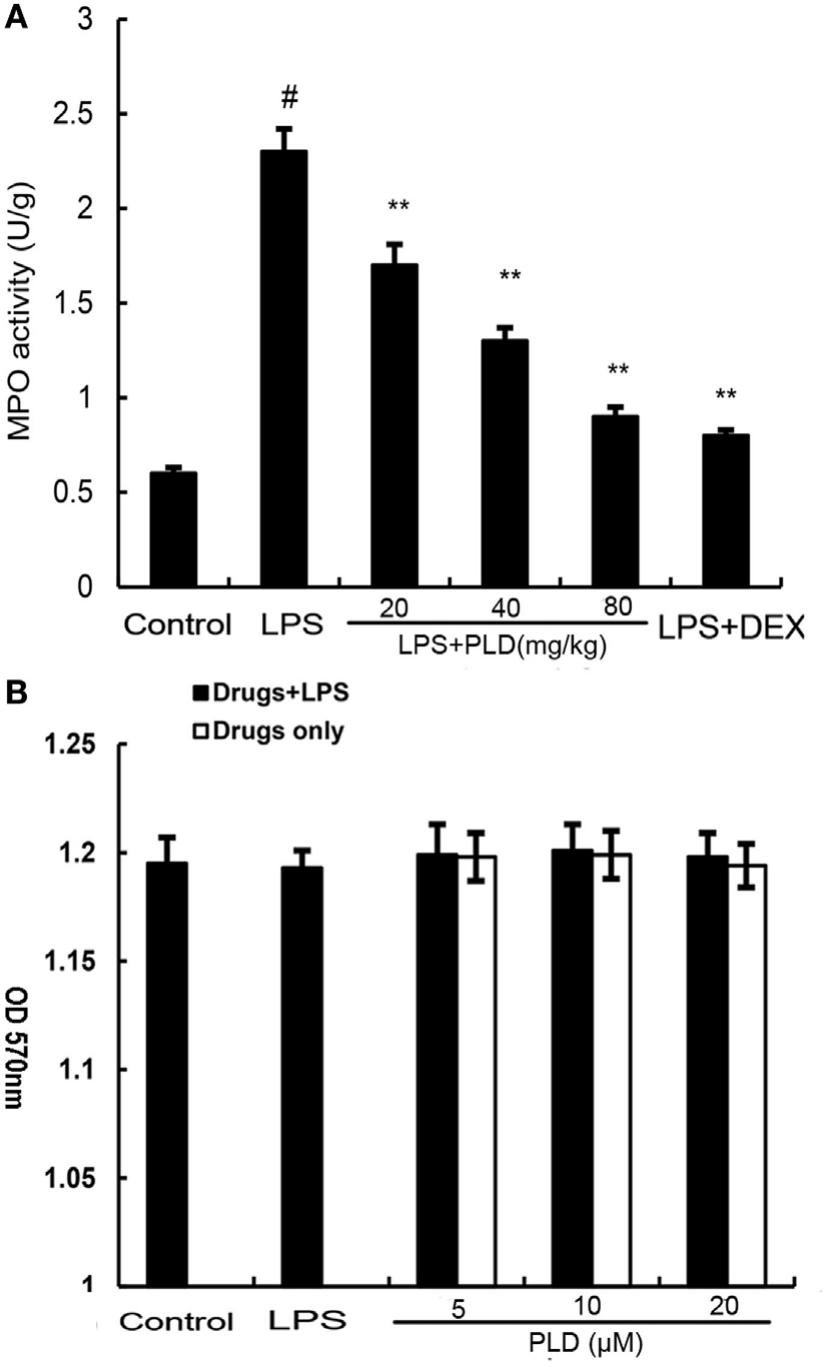
**(A)** Effects of platycodin D (PLD) on myeloperoxidase (MPO) activity in lung tissues of lipopolysaccharide (LPS)-induced ALI. MPO activity was determined at 7 h after LPS administration. **(B)**. Effect of PLD on the cell viability of A549 lung epithelial cells. The cell viability was determined by MTT assay. The values presented are the means ± SEM of three independent experiments, and differences between mean values were assessed by one-way ANOVA (Dunnett’s *t*-test) and the two-tailed Student’s *t*-test. ^#^*p* < 0.01 vs. control group, *p < 0.05, **p < 0.01 vs. LPS group.

PMNs are the major components of inflammatory and immunological reactions in injured lungs. MPO activity served as a marker of PMN accumulation in the lung. As shown in Figure 3A, lung MPO activity was upregulated after LPS treatment compared with control group (*p* < *0.01*). However, PLD (20, 40, ad 80 mg/kg) (*p* < 0.01) and DEX (5 mg/kg) (*p* < 0.01) treatment prevented elevated MPO activity after LPS stimulation.

### Effects of PLD on Cell Viability

MTT assay was used to test the effect of PLD on the potential cytotoxicity of cell. As shown in Figure 3B, cell viabilities were not affected by the PLD (5, 10, and 20 µM) at concentrations used. Thus, the effects of PLD on A549 cells were not attributable to cytotoxic effects.

### Effects of PLD on LPS-Induced Cytokine Production

**Figure 4:**
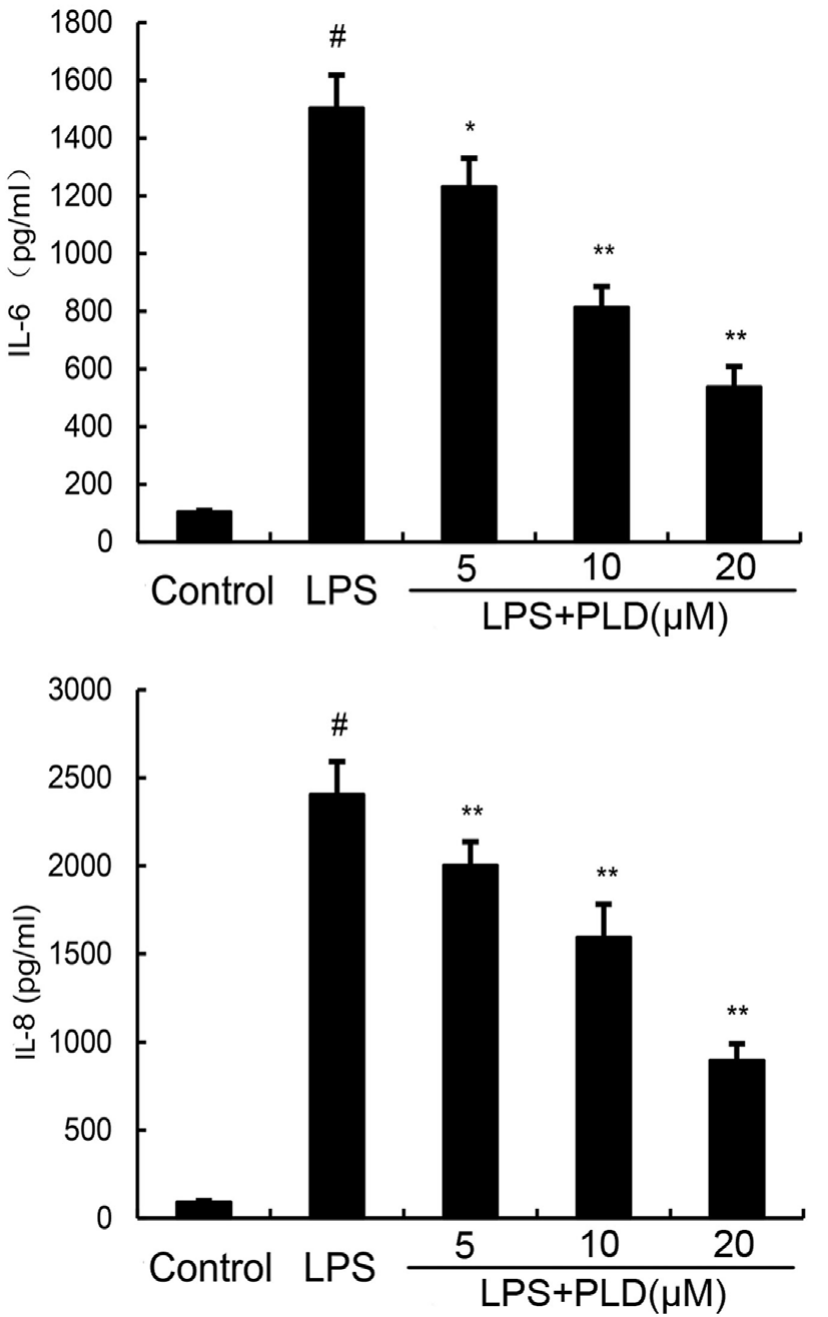
**Platycodin D inhibits lipopolysaccharide (LPS)-induced cytokine production in a dose-dependent manner**. Levels of IL-6 and IL-8 in culture supernatants were measured by ELISA. The data presented are the means ± SEM of three independent experiments, and differences between mean values were assessed by ANOVA. ^#^*p* < 0.05 vs. control group; *p < 0.05, **p < 0.01 vs. LPS group.

To investigate the protective effects of PLD *in vitro*, the production of IL-6 and IL-8 in the culture supernatants of A549 cells were measured by ELISA. As shown in Figure 4, the production of IL-6 and IL-8 increased after LPS stimulation. However, PLD significantly suppressed IL-6 and IL-8 production in LPS-stimulated A549 cells (Figure 4).

### Effects of PLD on LPS-Induced NF-κB and IRF3 Activation

**Figure 5:**
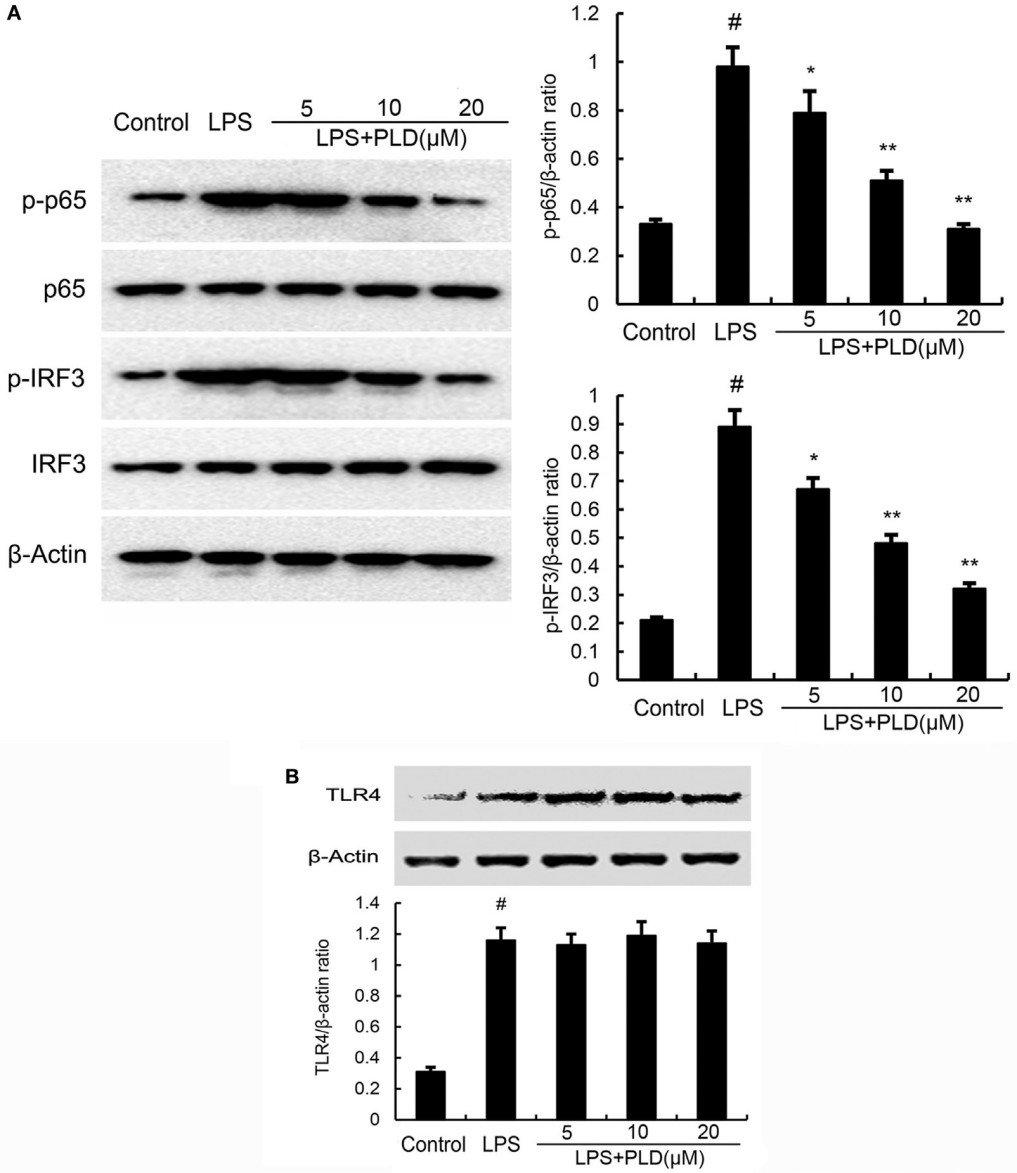
**(A)** Platycodin D (PLD) inhibits lipopolysaccharide (LPS)-induced NF-κB and IRF3 activation. A549 cells were preincubated with PLD (5, 10, and 20 µM) for 1 h and then treated with 1 µg/ml LPS for 1 h. **(B)** PLD does not affect TLR4 expression. Cells were preincubated with PLD (5, 10, and 20 µM) for 1 h and then treated with 1 µg/ml LPS for 6 h. Protein samples were analyzed by western blot with specific antibodies. The gels have been run under the same experimental conditions. The data presented are the means ± SEM of three independent experiments, and differences between mean values were assessed by one-way ANOVA and the two-tailed Student’s *t*-test. ^#^*p* < 0.05 vs. control group; *p < 0.05, **p < 0.01 vs. LPS group.

To test whether the anti-inflammatory effect of PLD is mediated through the NF-κB and IRF3 pathway, we measured the expression of NF-κB and IRF3 protein by Western blotting. As shown in Figure 5A, LPS significantly induced NF-κB and IRF3 activation in A549 cells. However, PLD significantly inhibited NF-κB and IRF3 activation induced by LPS.

### PLD Does Not Act through Affecting the Expression of TLR4

TLR4 is the upstream molecule of NF-κB and IRF3. Meanwhile, TLR4 is the major receptor of LPS. Therefore, we investigated whether PLD inhibited LPS-induced inflammatory response by suppressing TLR4 expression. As shown in Figure 5B, the results showed that LPS significantly elevated the expression of TLR4. PLD did not affect the expression of TLR4 upregulated by LPS (Figure 5B).

### PLD Inhibits Translocation of TLR4 to Lipid Rafts

**Figure 6:**
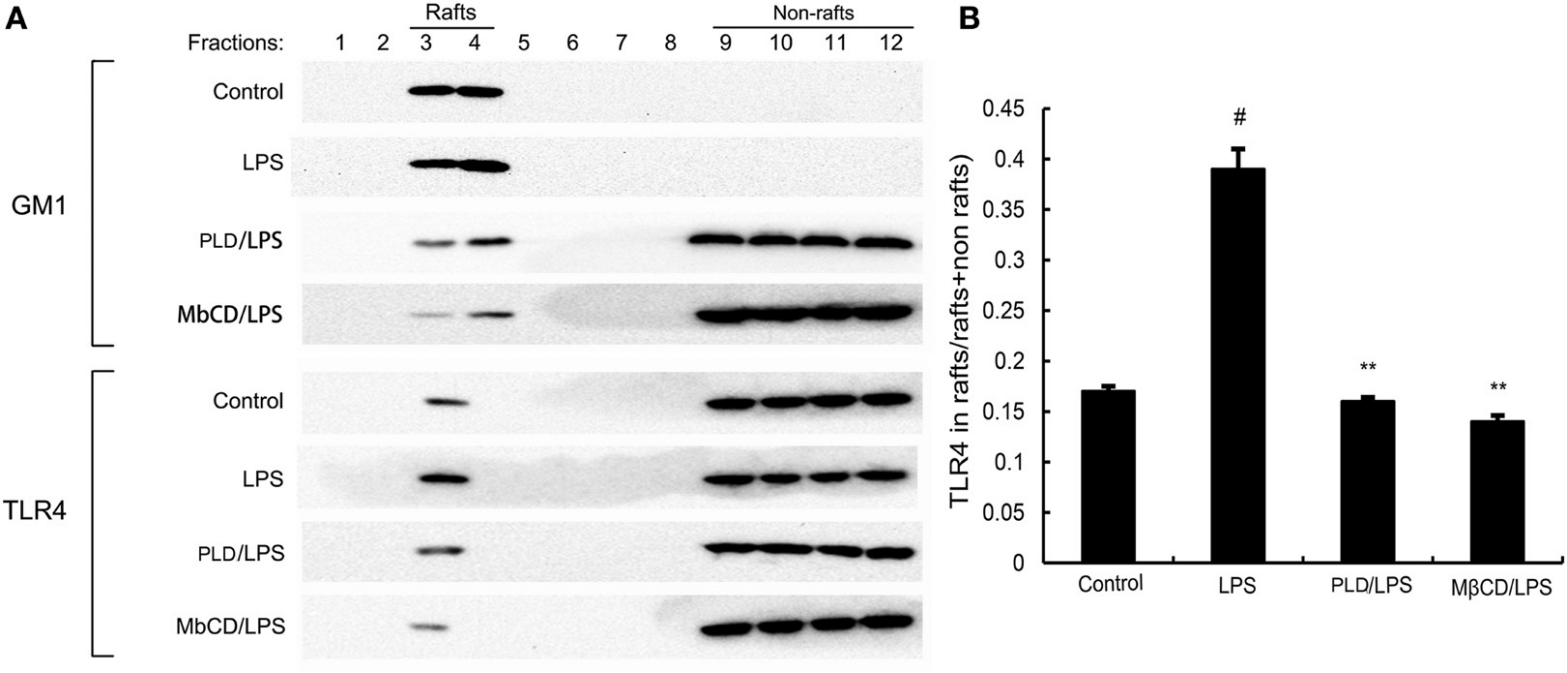
**The recruitment of TLR4 to lipid rafts was inhibited by platycodin D (PLD)**. **(A)** A549 cells were pretreated with PLD or MβCD, followed by treatment with 1 μg/ml lipopolysaccharide (LPS). The cells were lysed and subjected to discontinuous sucrose density gradient centrifugation as described in Section “Materials and Methods.” Fractions 3–4 correspond to lipid rafts. Representative blots of six separate experiments are shown. Fractions 9–12 correspond to non-lipid rafts. **(B)** TLR4 content of macrophage lipid rafts was calculated as a percentage of total membrane TLR4 (lipid rafts + non-rafts). The values presented are the means ± SEM of three independent experiments, and differences between mean values were assessed by Students’s *t*-test. The gels have been run under the same experimental conditions. ^#^*p* < 0.05 vs. control group; *p < 0.05, **p < 0.01 vs. LPS group.

Lipid rafts played an important role in LPS/TLR4 signaling pathway. Stimulating cells with LPS could induce TLR4 recruit to lipid rafts. To further address the potential anti-inflammatory mechanism of PLD, we determined the effects of PLD on the recruitment of TLR4 into lipid rafts. We isolated raft fractions and examined the translocation of TLR4 by immunoblotting. The results showed that LPS stimulation induced localization of TLR4 to raft fractions. PLD or MβCD reduced TLR4 recruitment into lipid rafts (Figure 6).

### PLD Disrupts the Formation of Lipid Rafts in Cell Membranes by Depleting Cholesterol

**Figure 7:**
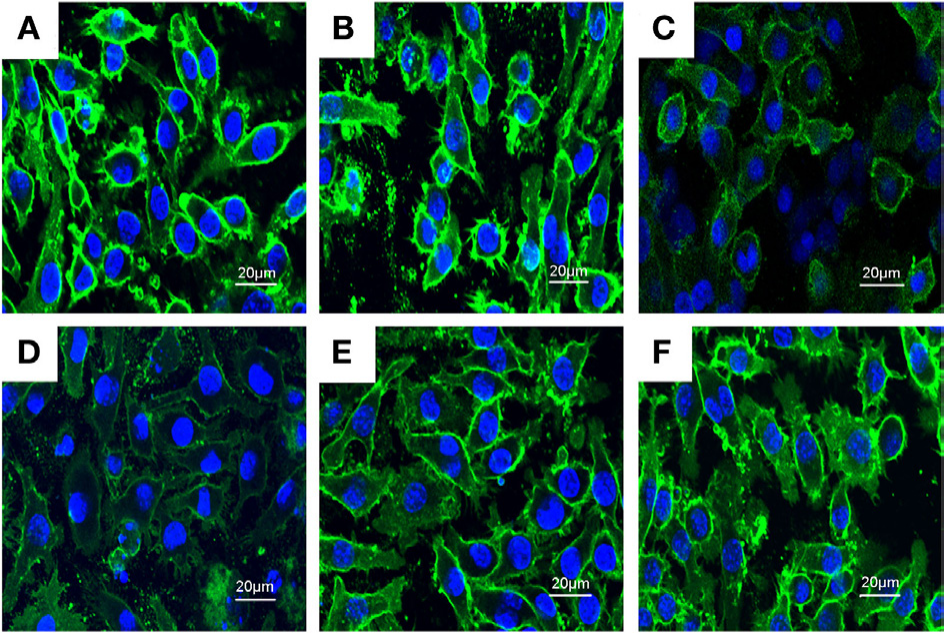
**The inhibition of the formation of lipid rafts by platycodin D (PLD)**. The cells were preincubated with PLD (5, 10, and 20 µM) for 1 h, followed by treatment with 1 µg/ml lipopolysaccharide (LPS) for 6 h. The lipid rafts (green) were stained with Alexa Flour 488-conjugated CTxB, and the nucleus was stained with Hochest. **(A)** Control group, **(B)** LPS group, **(C)** LPS + MβCD group, **(D)** LPS + PLD 12 µM, **(E)** LPS + PLD 6 µM, and **(F)** LPS + PLD 3 µM.

**Figure 8:**
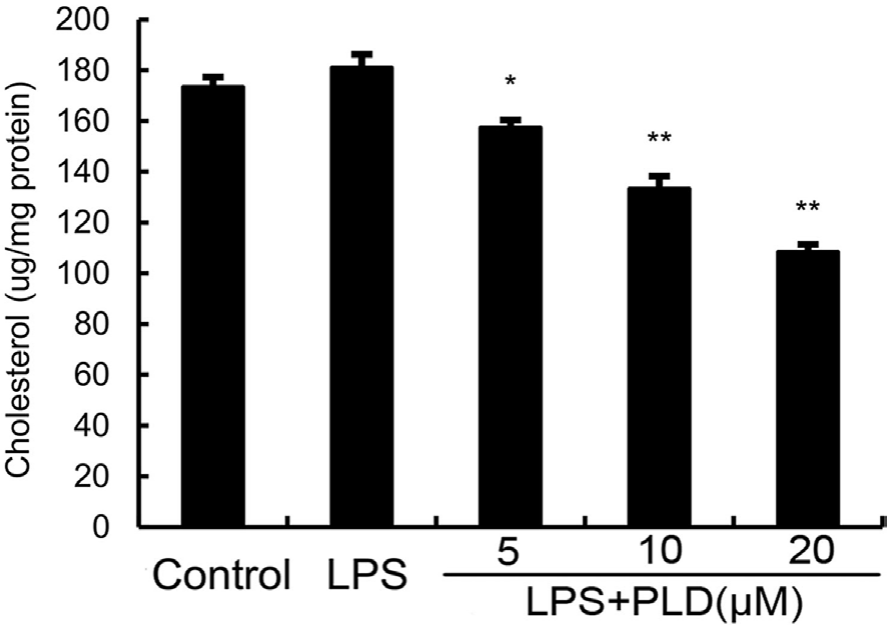
**Effects of platycodin D (PLD) on membrane lipid rafts cholesterol levels**. A549 cells were treated with PLD (5, 10, and 20 µM) for 12 h. Membrane cholesterol levels were measured by gas–liquid chromatography, and the results were plotted as microgram cholesterol per milligram protein. The values presented are the means ± SEM of three independent experiments, and differences between mean values were assessed by Students’s *t*-test (*p < 0.05, **p < 0.01).

Previous studies showed that inhibition the formation of lipid rafts could prevent translocation of TLR4 to lipid rafts. GM1 has been known to be a marker of lipid rafts. To investigate the anti-inflammatory mechanism of PLD, the formation of lipid rafts were tested by assessing GM-1 expression by confocal laser microscopy. PLD disrupted the formation of lipid rafts by depleting cholesterol (Figure 7). Meanwhile, the results showed that PLD disrupted the lipid rafts by removing of cholesterol from lipid rafts in a dose-dependent manner (Figure 8).

### Cholesterol Replenishment Prevents the Anti-inflammatory Effect of PLD

**Figure 9:**
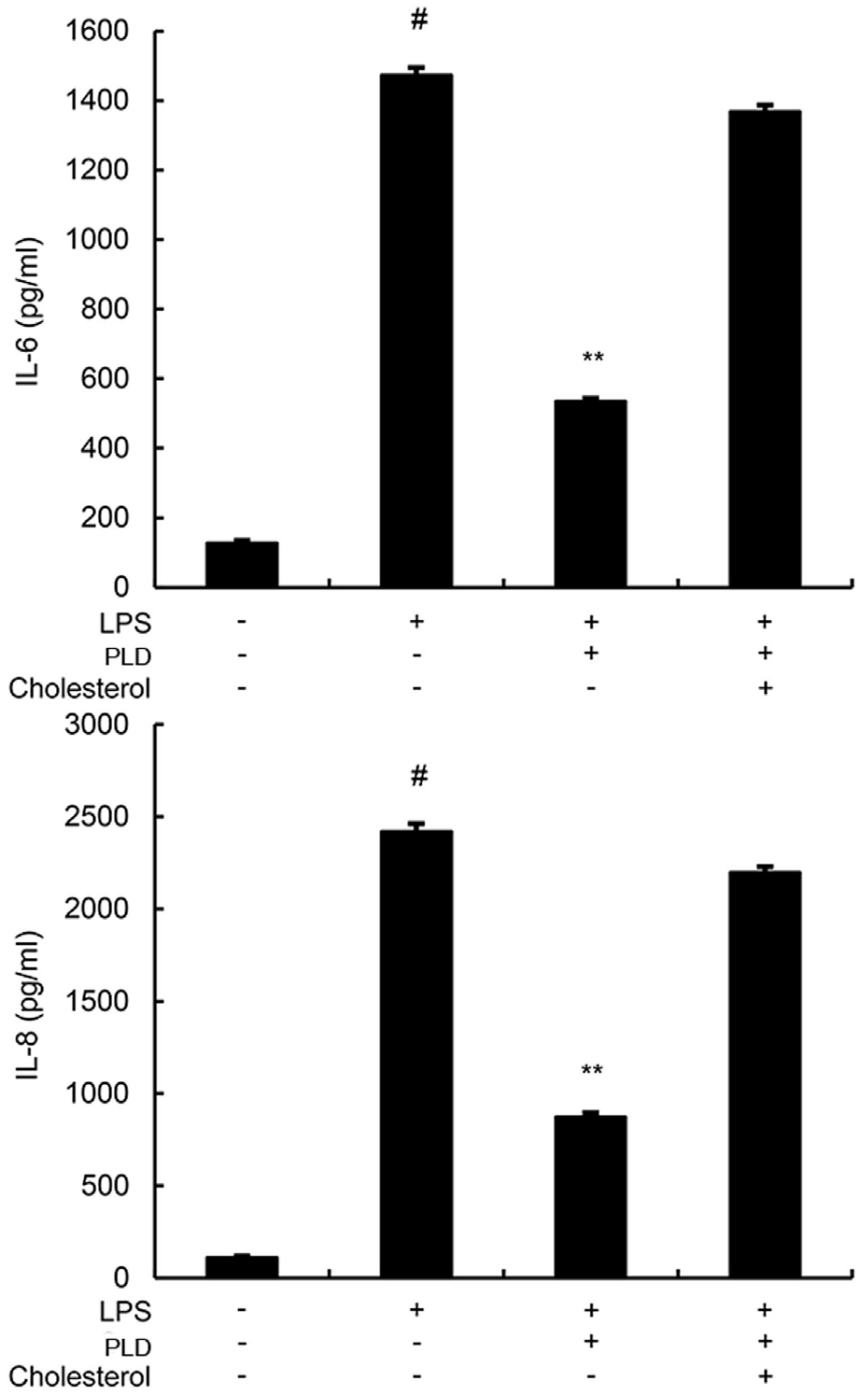
**A549 cells were treated with culture medium alone or medium containing platycodin D (20 µM), or MβCD (10 mM) at 37°C for 60 min**. Subsequently, the cells were washed with PBS and incubated with medium alone or medium containing water-soluble cholesterol (84 µg/ml) for 30 min. Cells were treated with 1 µg/ml lipopolysaccharide (LPS) for 24 h. Levels of IL-6 and IL-8 in culture supernatants were measured by ELISA. The data presented are the means ± SEM of three independent experiments, and differences between mean values were assessed by ANOVA. ^#^*p* < 0.05 vs. control group; *p < 0.05, **p < 0.01 vs. LPS group.

Cholesterol replenishment experiments were used to clarify the mechanism of PLD. As shown in Figure 9, the inhibition effect of PLD on LPS-induced cytokines production was abolished.

### PLD Activates LXRα and ABCA1

**Figure 10:**
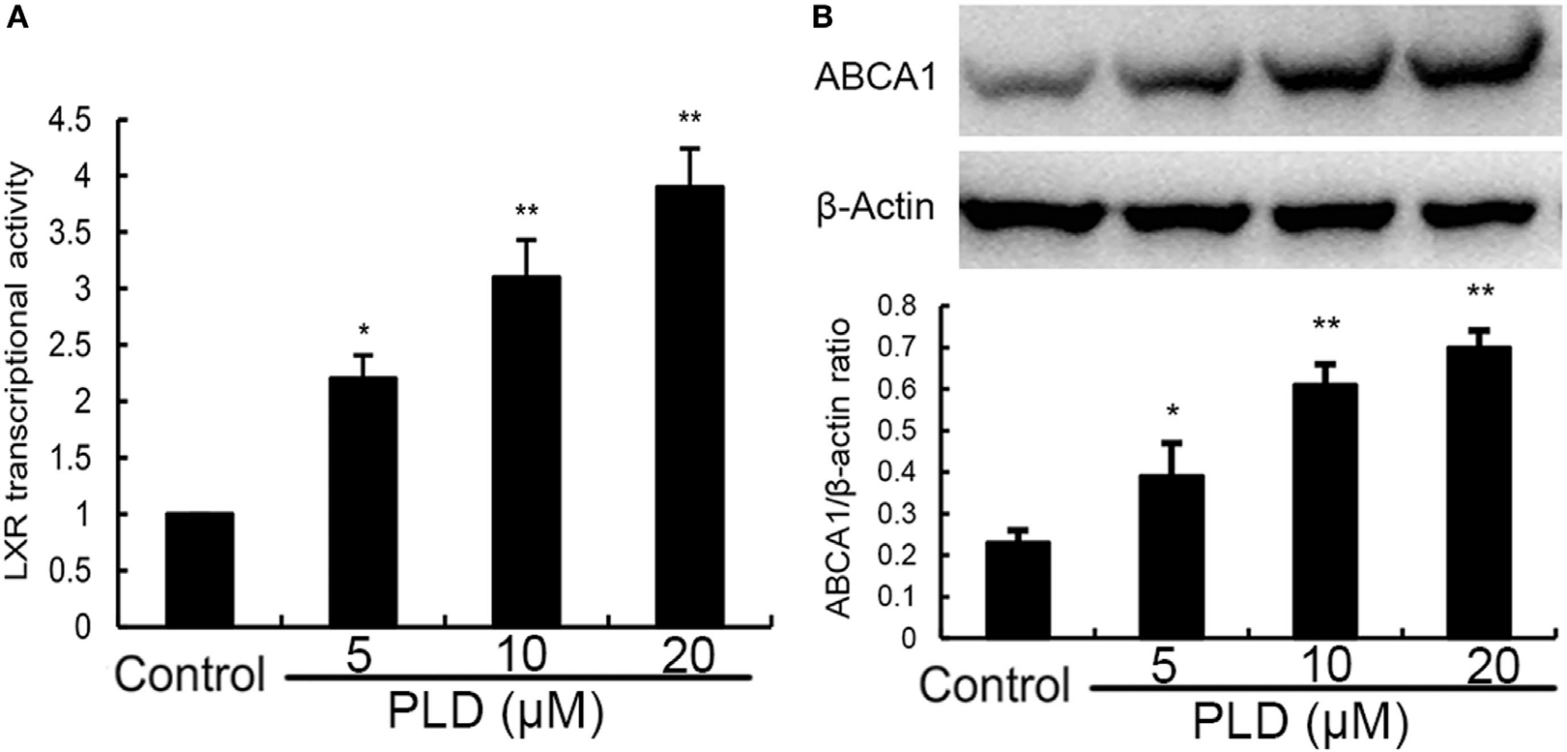
**(A)** Effects of platycodin D (PLD) on LXRα transcriptional activity. Relative luciferase activity was determined by normalization with β-galactosidase activity. The data are normalized to control group (*p < 0.05, **p < 0.01). **(B)** Effects of PLD on ABCA1 expression. The values presented are the means ± SEM of three independent experiments, and differences between mean values were assessed by Students’s *t*-test (*p < 0.05, **p < 0.01). The gels have been run under the same experimental conditions.^#^ *p* < 0.05 vs. control group; *p < 0.05, **p < 0.01 vs. lipopolysaccharide group.

Activation of LXRα could regulate intracellular cholesterol levels. Thus, we performed a luciferase reporter gene assay to test whether PLD could enhance transcriptional activity of LXRα. As shown in Figure 10A, PLD dose-dependently increased expression of the LXR luciferase reporter gene. Meanwhile, the expression of ABCA1 was detected by Western blot analysis. As shown in Figure 10B, PLD upregulated the expression of ABCA1 in a dose-dependent manner.

### Knockdown of LXRα Abrogated the Effects of PLD on LPS Induced Inflammatory Response

**Figure 11:**
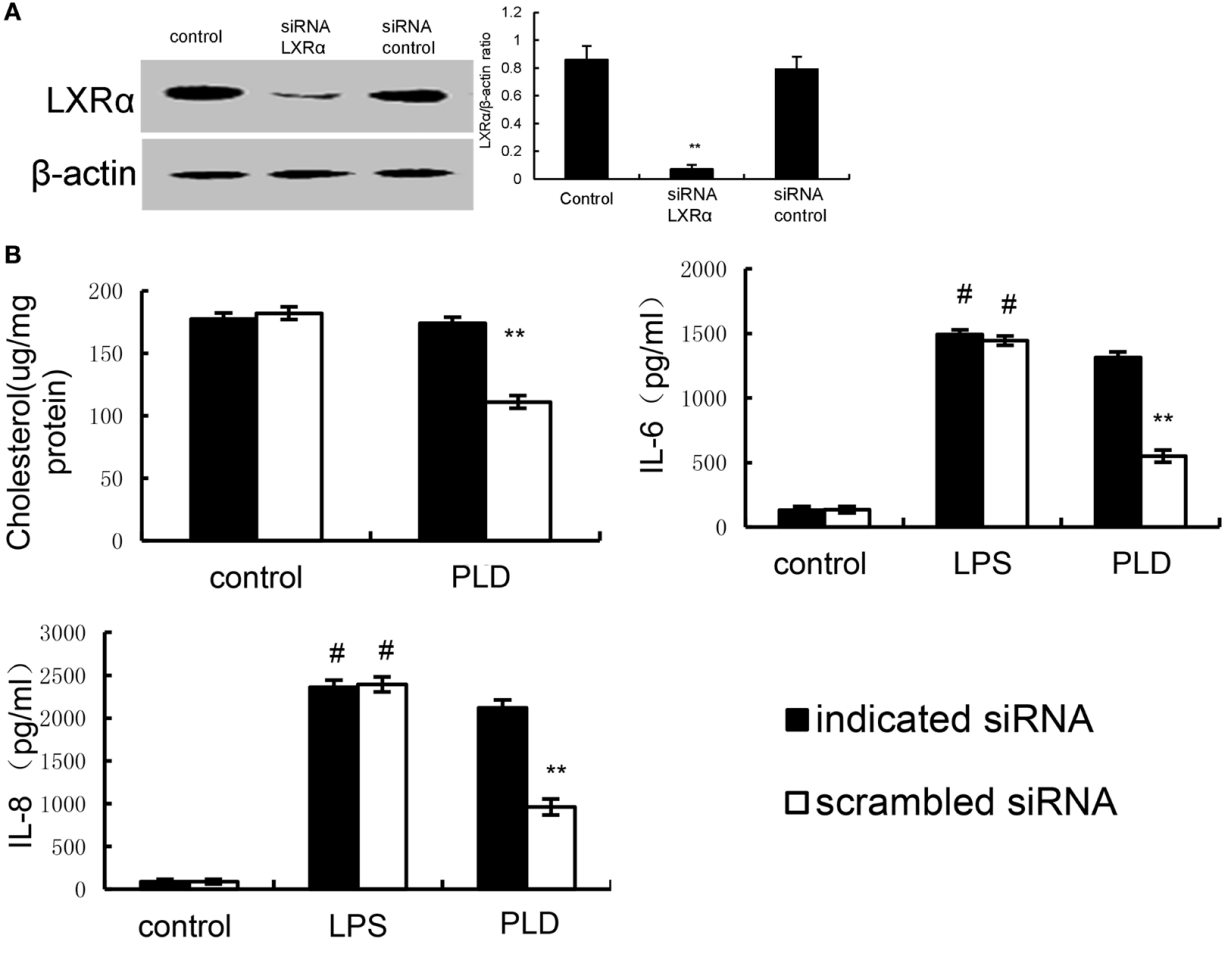
**(A)** Cells were transfected with a siRNA specific for LXRα, or a scrambled siRNA (negative control) as indicated. The effects of siRNA on LXRα expression was detected by Western blot analysis. **(B)** Knockdown of LXRα abrogated the effects of Platycodin D on lipid rafts cholesterol levels, and lipopolysaccharide (LPS) induces inflammatory response. A549 cells were transfected with a siRNA specific for LXRα, or a scrambled siRNA (negative control) as indicated. Lipid raft cholesterol levels were detected. Levels of IL-8 and IL-6 in culture supernatants were measured by ELISA. The data presented are the means ± SEM of three independent experiments, and differences between mean values were assessed by Students’s *t*-test. ^#^*p* < 0.05 vs. control group; *p < 0.05, **p < 0.01 vs. LPS group.

We tested whether the anti-inflammatory mechanism of PLD is through LXRα, LXRα was silenced using specific siRNA. The results showed that the expression of LXRα was significantly inhibited by siRNA. Once LXRα was knockdown, the effects of PLD on cholesterol levels and cytokines production induced by LPS were reversed (Figure 11).

## Discussion

Acute lung injury, or its severe form ARDS, is characterized by the release of inflammatory mediators (14). Previous study showed that PLD could protect against airway inflammation (10). In this study, our results showed that PLD significantly attenuated LPS-induced lung damage. *In vitro*, PLD inhibited the production of cytokines by reducing TLR4 migration into lipid rafts in LPS-activated A549 lung epithelial cells.

Myeloperoxidase activity is assessed for the quantification of neutrophil accumulation in tissues (15). In this study, we found that treatment with PLD significantly decreased LPS-induced increases in MPO activity in the lung tissues compared with the LPS group. Histologic observation demonstrated marked thickening of the alveolus walls and significant infiltration of inflammatory cells in LPS-induced ALI. Administration of PLD remarkably attenuated lung pathological changes. These results showed that PLD had a protective effect against LPS-induced ALI.

The lung epithelial cells provide a physical barrier and play an important role in defense against invading microbial pathogens (16). The lung epithelial cells recognize various pathogens and respond by secreting chemokines and cytokines to alert innate and adaptive immune system to prevent and control infection. Studies showed that stimulation of lung epithelial cells by LPS could induce TNF-α, IL-8, and IL-6 production (17, 18). These cytokines and chemokines play important roles in the progress of ALI (19). In this study, our results showed that PLD dose-dependently inhibited IL-8, and IL-6 production in LPS-stimulated A549 lung epithelial cells.

Activation of TLR4 by LPS leads to NF-κB and IRF3 activation and cytokines release (20). NF-κB and IRF3 play important roles in regulating inflammatory cytokines production. To test the inhibitory mechanism of cytokines production, the effects of PLD on NF-κB and IRF3 activation were measured. Our results suggested that PLD could inhibit LPS-induced NF-κB and IRF3 activation in a dose-dependent manner. This was consistent with previous study which showed PLD protected against OVA-induced asthma by inhibiting NF-κB activation (10). The effect of PLD on TLR4 expression was tested to investigate the mechanism of PLD. The results showed that PLD did not inhibit TLR4 expression.

Lipid rafts are detergent insoluble glycolipid-enriched membrance domains. Lipid rafts play a critical role in TLR4 signaling pathway (21). In this study, our results demonstrated that PLD inhibited translocation of TLR4 to lipid rafts (Figure 8). Meanwhile, the results in Figures 8 and 9 showed that PLD disrupted the formation of lipid rafts by depleting cholesterol. Studies showed that treatment with raft-disrupting drugs (depleting cholesterol) could inhibit TLR4 translocation into lipid rafts and LPS induced NF-κB activation and TNF-α production (6, 7). It is suggested that PLD disrupts lipid rafts by depleting cholesterol. And the disruption of lipid rafts leads to the inhibition of TLR4 translocation to lipid raft in A549 lung epithelial cells. Cholesterol replenishment prevents the anti-inflammatory effect of PLD.

LXRs regulate intracellular cholesterol levels through ATP-binding cassette transporter A1 (ABCA1). ABCA1, located in the membrane of the cells, is a plasma membrane protein. It plays a critical role in the regulation of cholesterol (22). ABCA1 inhibits TLR4 trafficking to lipid rafts by decreasing the level of cholesterol in lipid rafts (23). To investigate the anti-inflammatory mechanism of PLD, the effects of PLD on LXRα activation were measured. As shown in the results, PLD could activate LXRα–ABCA1 pathway. These results indicated that PLD activated LXRα–ABCA1 pathway by mediating cholesterol efflux to reduce lipid rafts cholesterol content in A549 lung epithelial cells.

In conclusion, our results demonstrated that PLD inhibited inflammatory cytokines production in LPS-stimulated A549 lung epithelial cells. The promising anti-inflammatory effect of PLD is associated with upregulation of the LXRα–ABCA1 pathway which results in disrupting lipid rafts by depleting cholesterol and reducing translocation of TLR4 to lipid rafts, thereby suppressing LPS-induced inflammatory response. PLD may have been used as an anti-inflammatory agent for lung inflammation and other inflammatory diseases.

## Ethics Statement

All animal experiments were performed in accordance with the guide for the Care and Use of Laboratory Animals published by the US National Institute of Health.

## Author Contributions

XH contributed to article writing, literature search, and results evaluation. XL performed histologic analysis and article revision. WZ and YC performed the final revision of the article and expert opinions. ZZ contributed to literature search and results evaluation. YF performed the final revision of the article and results evaluation. NZ contributed to study design.

## Conflict of Interest Statement

The authors declare that the research was conducted in the absence of any commercial or financial relationships that could be construed as a potential conflict of interest.
